# Global burden of HIV, syphilis, and HBV infection among women of childbearing age and children under five: based on the Global Burden of Diseases Study 2021

**DOI:** 10.3389/fpubh.2025.1666408

**Published:** 2025-10-16

**Authors:** Jia Lian, Bihong Ke, Xiaosheng Zhang, Jinbo Li, Yandi Li, Keke Wang, Yongliang Feng, Suping Wang

**Affiliations:** ^1^Department of Epidemiology, School of Public Health, Shanxi Medical University, Taiyuan, China; ^2^Center of Clinical Epidemiology and Evidence Based Medicine, Shanxi Medical University, Taiyuan, China; ^3^MOE Key Laboratory of Coal Environmental Pathogenicity and Prevention, Shanxi Medical University, Taiyuan, China; ^4^Research Center for Reverse Microbial Etiology, Workstation of Academician, Shanxi Medical University, Taiyuan, China; ^5^First Hospital of Shanxi Medical University, Taiyuan, China

**Keywords:** global disease burden, women of child-bearing age, children under five, HIV, syphilis, HBV infection

## Abstract

**Introduction:**

Human immunodeficiency virus (HIV), syphilis, and hepatitis B virus (HBV) infection pose a major global public health challenge. However, high-quality integrated data on the burden of these diseases among women of childbearing age (WCBA) and children remain limited. This study aims to provide a comprehensive global assessment of HIV, syphilis, and HBV infection among WCBA and children under five and to explore their potential relationships.

**Methods:**

The Global Burden of Disease Study 2021 provided data on the global prevalence of WCBA and incidence of HIV, syphilis, and HBV infection in children under five years old from 1990 to 2021. Temporal trends were analyzed using annual average percentage change (AAPC) and joinpoint regression. A Bayesian age-period-cohort model was used to forecast the disease burden from 2022 to 2030.

**Results:**

In 2021, the age-standardized prevalence (ASPR) of HIV infection among WCBA was 830.87 per 100,000, while the age-standardized incidence (ASIR) of HIV among children under five was 15.39 per 100,000. From 1990 to 2021, the ASPR of HIV in WCBA demonstrated sustained growth, whereas the ASIR in children exhibited a rise-then-fall pattern. For syphilis, the 2021 ASPR among WCBA was 1057.32 per 100,000, and the ASIR in children was 58.36 per 100,000; both metrics followed a declining-then-rising trend, returning to 1990 levels by 2021. Both the ASPR of HBV infection among WCBA and the ASIR among children under five declined significantly from 1990 to 2021, with average annual percentage changes of −1.25% and −4.14%, respectively. Generally, ASPR and ASIR decreased as the sociodemographic index (SDI) increased. Regions with lower SDI experienced a disproportionately higher burden of HIV, syphilis, and HBV infections.

**Conclusion:**

In 2021, the global disease burden of HIV, syphilis, and HBV infection remained high among WCBA and children under five, with significant regional and demographic disparities. These findings indicate a persistent public health challenge, necessitating continued global efforts to mitigate the burden through addressing socioeconomic inequalities, strengthening health education, and expanding healthcare access.

## 1 Introduction

Adolescent girls and young women (AGYW) constitute a vital demographic with significant social and economic potential. As a sexually active population, they face a higher risk of sexually transmitted infections (STIs). Decisions made during adolescence exert immediate and long-term effects on future economic opportunities, health trajectories, and skill acquisition. Women of child-bearing age (WCBA), defined as those aged 15–59 years, represent a critical population for reproductive health. Their infection status directly influences mother-to-child transmission (MTCT) rates and perinatal outcomes (1). AGYW are a key subgroup within this broader WCBA demographic. Human immunodeficiency virus (HIV), syphilis, and hepatitis B virus (HBV) infection are significant sexually transmitted diseases and also represent major causes of MTCT. This transmission can occur during pregnancy, delivery, or breastfeeding, leading to substantial maternal and child morbidity, mortality, and long-term disease burden.

Epidemiological evidence indicates alarming transmission risks in untreated cases. Without intervention, the MTCT rate for HIV ranges from 15 to 45% (2). Maternal HIV infection increases child mortality both directly and indirectly through associated complications. Similarly, neonates exposed to HBV face infection rates of 70–90% when born to HBsAg- and HBeAg-positive mothers and 5–20% for HBsAg-positive and HBeAg-negative mothers (3). An estimated 90% of perinatally infected newborns develop chronic HBV infection and subsequent liver diseases, such as cirrhosis and hepatocellular carcinoma, far exceeding the 5% chronicity rate in adults. Untreated maternal syphilis is equally devastating, causing adverse pregnancy outcomes in approximately 70% of cases, including fetal loss, stillbirth, congenital syphilis, or neonatal death (4).

HIV, syphilis, and HBV infection share common transmission routes, leading to frequent co-infections and complex interactions (5). They are also driven by common social and structural determinants, including socio-economic inequality, limited access to education and sexual health services, and gender-based violence. Biologically, active syphilis infection increases susceptibility to HIV infection (6), and HBV/HIV coinfection accelerates disease progression, resulting in a higher risk of liver deterioration than HBV mono-infection. Notably, HIV, syphilis, and HBV co-infections exhibit synergistic interactions that exacerbate disease progression and amplify MTCT risks, further complicating clinical management (7). These findings underscore the urgent need to integrate epidemiological surveillance with targeted MTCT prevention strategies, emphasizing the lifelong health implications of perinatal infections.

Triple elimination refers to the initiative to eliminate the MTCT of HIV, syphilis, and HBV infection prevalent in low- and middle-income countries. This initiative aligns with the World Health Organization’s (WHO) Global Health Sector Strategies (GHSS) on HIV, viral hepatitis, and STDs for 2022–2030 (8), supporting broader goals such as the Global Alliance to End AIDS in Children (9) and the GHSS target to eliminate viral hepatitis as a public health threat by 2030. Furthermore, the 69th World Health Assembly in 2016 set a 90% reduction in global syphilis incidence by 2030 (10). Given the interconnectedness of these diseases, HBV vaccination and syphilis screening for WCBA are also effective HIV prevention strategies. It is essential to estimate the disease burden of these infections among WCBA and children under five at national, regional, and sociodemographic index (SDI) levels. Such data are crucial for informing targeted interventions and accelerating progress toward triple elimination.

In this study, we utilized the Global Burden of Disease, Injuries, and Risk Factors Study (GBD) 2021 database (11) to analyze the disease burden of HIV, syphilis, and HBV infection among WCBA and children under five globally and across subnational regions. By analyzing these epidemiological metrics, we aim to elucidate the association between maternal infections and adverse pediatric outcomes, highlighting the preventable burden of MTCT-related morbidity and mortality, and inform evidence-based policy frameworks to reduce perinatal transmission and alleviate chronic health consequences in affected children. The primary significance of this work lies in providing actionable insights to refine MTCT prevention strategies, optimize resource allocation, and advance progress toward triple elimination goals.

## 2 Methods

### 2.1 Data sources

The data for this study were obtained from the GBD study 2021, which analyzed the disease burden of an estimated 371 diseases and injuries across 204 countries and territories (11). We extracted data on incidence, prevalence, disability-adjusted life-years (DALYs), age-standardized incidence (ASIR), age-standardized prevalence (ASPR), and age-standardized DALY rate of WCBA and children under five. Age-specific and country-specific estimates were generated using the Global Health Data Exchange Query Tool^1^ (12). The disease burden attributable to HBV infection included acute HBV infection, HBV-induced liver cirrhosis, and hepatocellular carcinoma (HCC) secondary to chronic HBV infection.

The SDI, developed by GBD researchers and calculated for GBD study 2021, was used to assess the combined level of health-related social and economic development across countries and territories. The SDI is a composite indicator (ranging from 0 to 1) that incorporates a country’s lag-distributed income per capita, average years of schooling, and total fertility rate among females under 25 years. Based on the country-level SDI estimates for 2021, the 204 countries were categorized into quintiles: low, low-middle, middle, high-middle, and high.

### 2.2 Data analysis

As the included data were stratified by age groups and time period, the age-standardized rate (ASR) based on the world standard population in the GBD database was used for analysis. We used the direct standardization method to calculate ASRs for specific age groups using the “ageadjust.direct” function from the “epitools” package in R software (13–15). Heterogeneity was assessed using 95% uncertainty intervals (UIs). Joinpoint regression model was used to estimate the average annual percentage change (AAPC) and corresponding 95% confidence intervals (CI) for each segment (16). These models included both linear (y = xb) and log-linear (ln y = xb) forms. The log-linear model was applied to analyze population-based trends in ASRs in our study. The number and location of joinpoints were determined using the grid search method, allowing for a maximum of five joinpoints. Model optimization was performed using the Monte Carlo permutation test, following the methodology described in previous studies (17).

To predict the future ASRs for HIV, syphilis and HBV infection, we utilized the Bayesian age–period–cohort (BAPC) model to analyze the incidence, prevalence, and DALY rate of these three MTCT diseases from 2022 to 2030. A key advantage of the BAPC model is its implementation of the Integrated Nested Laplacian Approximation (INLA) method, which approximates the posterior distribution and helps avoid mixing and convergence problems associated with traditional Bayesian methods of Markov Chain Monte Carlo sampling (18). Due to its comprehensive coverage and ability to capture temporal trends, the BAPC model has been widely validated and applied in epidemiological research, particularly in studies involving age-structured population data and complex cohort effects (18).

Joinpoint regression analysis was performed using the Joinpoint Regression Program (version 5.0.2.0) (19). All data analyses and visualizations were conducted using R software (version 4.3.3). A *p*-value of < 0.05 (two-sided) was considered statistically significant.

## 3 Results

### 3.1 Global overview

**Figure 1:**
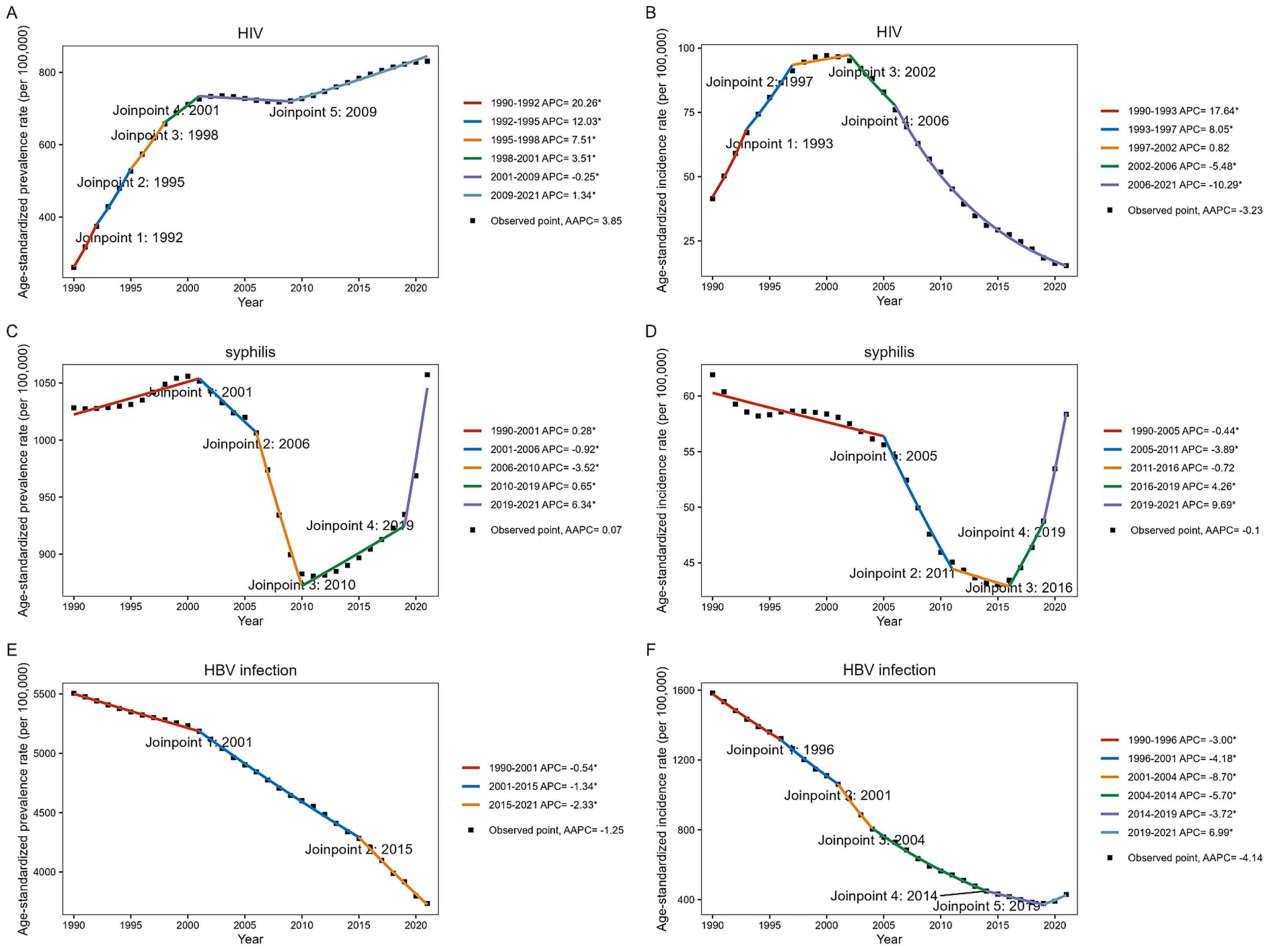
Joinpoint regression analysis of HIV, syphilis, and HBV infection prevalence among WCBA and incidence among children under 5 years globally, 1990–2021. **(A,C,E)** ASPR among WCBA. **(B,D,F)** ASPR among children under 5 years.

Globally, the number of prevalent HIV infection cases among WCBA increased dramatically from 3.55 million in 1990 to 16.49 million in 2021, representing a 364.51% increase over this period (Figure 1A; Supplementary file 1). The ASPR also increased significantly from 1990 to 2021, with an AAPC of 3.85% (95% CI: 3.48–4.22). Trend analysis revealed that the ASPR of HIV infection among WCBA increased markedly from 2001, stabilized during 2001 to 2009, and then gradually rose again in subsequent years. Conversely, the ASIR of HIV infection in children under 5 years declined by 3.32% between 1990 and 2021, reaching 15.39 per 100,000 in 2021. The ASIR in this age group increased until 2002, peaked in that year, and subsequently decreased steadily (Figure 1B; Supplementary file 2).

In 2021, there were 20.48 million prevalent cases of syphilis among WCBA worldwide. This represents a 103.98% increase from 1990, which had 10.04 million cases. Over the past 30 years, the ASPR of syphilis among WCBA initially increased, then decreased until approximately 2010, reached its lowest point that year, and subsequently increased annually, with an accelerated upward trend after 2019 (Figure 1C; Supplementary file 1). Among children under five, the ASIR of syphilis was 58.36 per 100,000 in 2021. The ASIR declined until 2016, reached its lowest level that year, and then increased sharply thereafter (Figure 1D; Supplementary file 2).

HBV infection remains a major global public health issue. The number of prevalent HBV infections among WCBA was estimated at 74.15 million in 1990 and 73.35 million in 2021. The ASPR from HBV infection showed a declining trend, with an AAPC of −1.25% (95%CI: −1.28 to −1.22) from 1990 to 2021 (Figure 1E; Supplementary file 1). Trend analysis revealed a sustained and significant decline in the ASPR for HBV infection among WCBA over the past three decades, both globally and across all SDI regions. Globally, an estimated 2.82 million new cases of HBV infection occurred among children under 5 years in 2021, corresponding to an ASIR of 429.60 per 100,000. Before 2019, this age group exhibited a consistent downward trend in HBV infection incidence, followed by a moderate increase (Figure 1F; Supplementary file 2).

### 3.2 Regional differences

**Figure 2:**
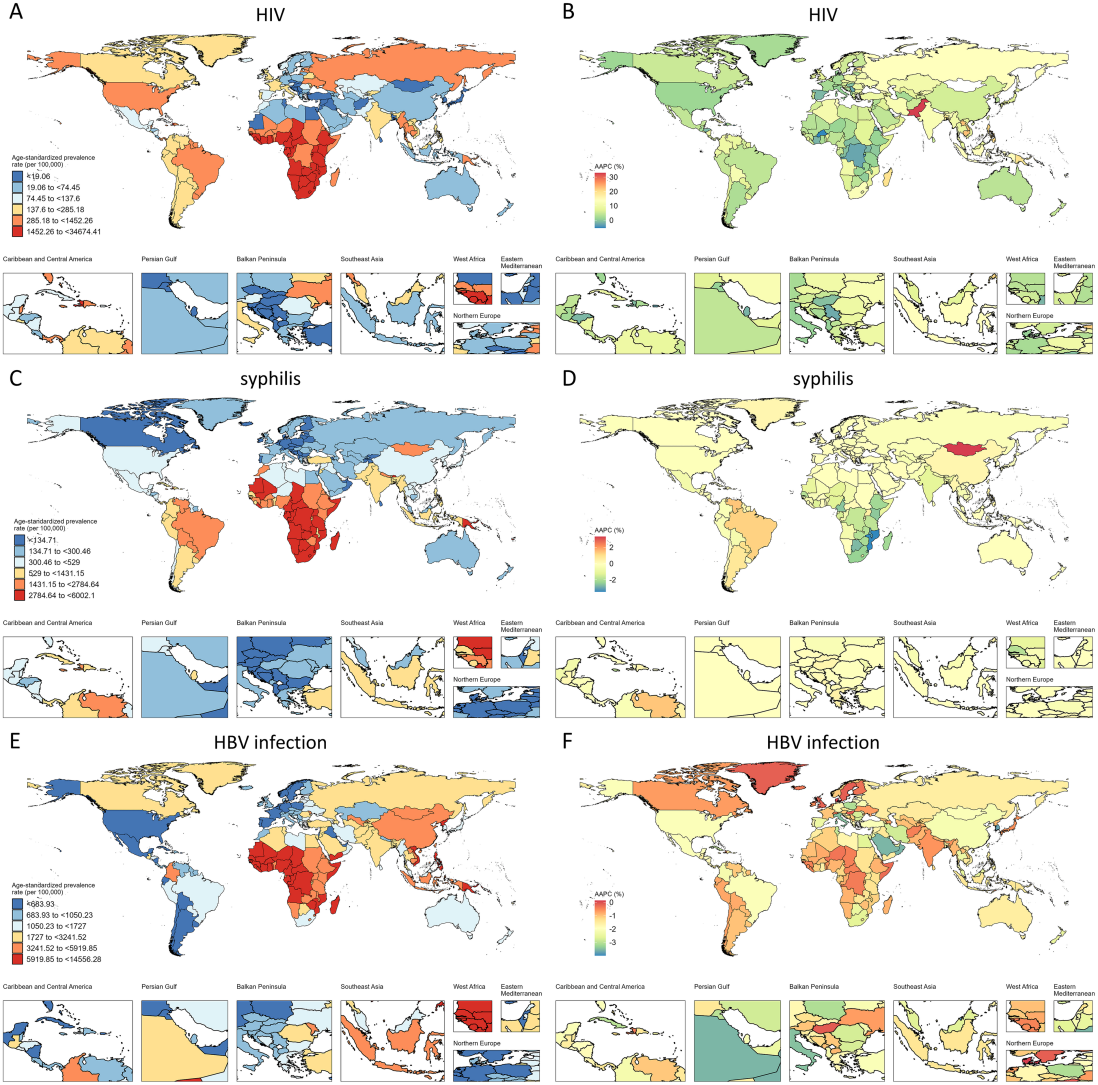
ASPR and AAPC of HIV, syphilis, and HBV infection among WCBA, by country. **(A,C,E)** ASPR in 2021. **(B,D,F)** AAPC from 1990 to 2021.

**Figure 3:**
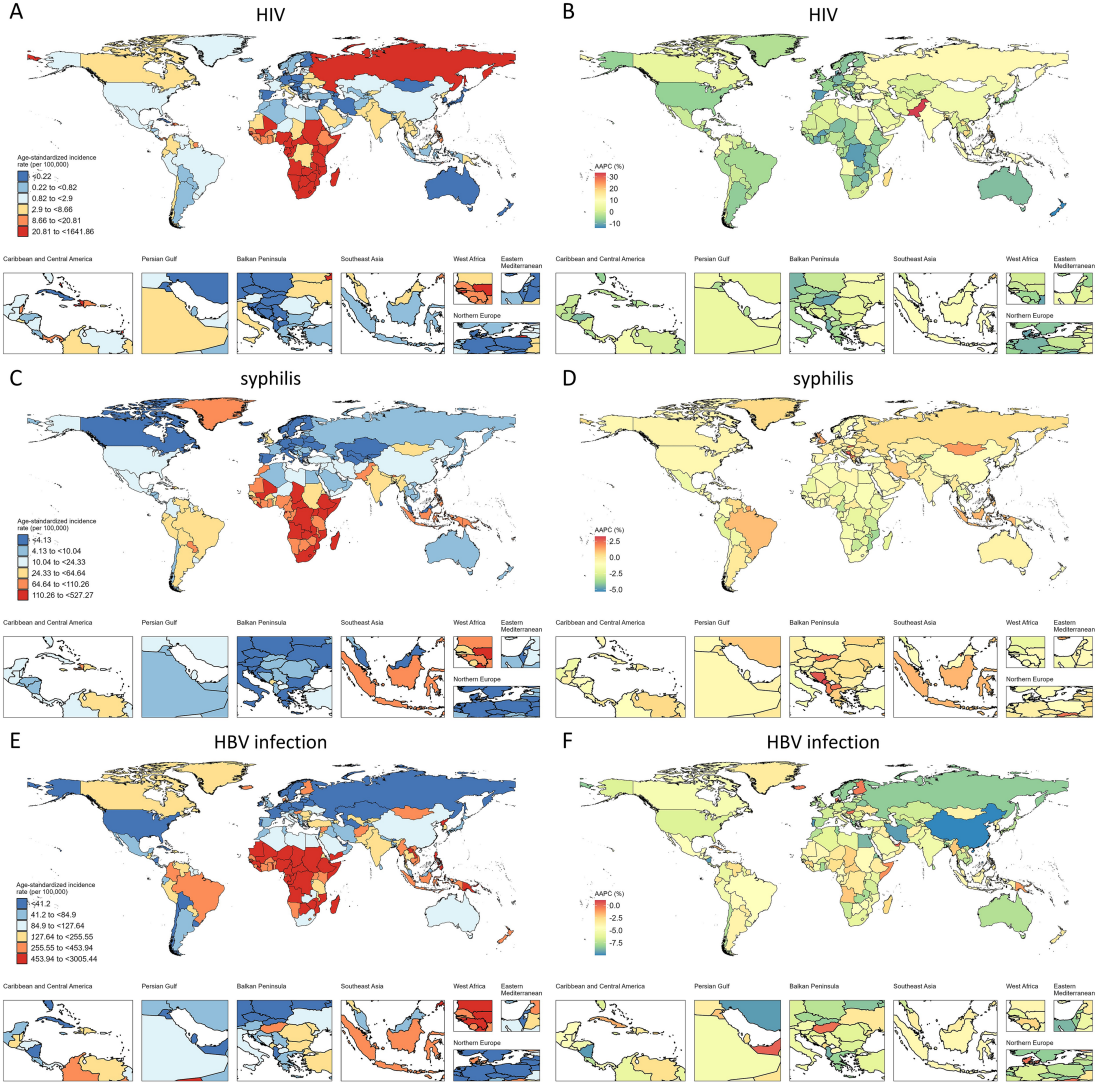
ASIR and AAPC of HIV, syphilis, and HBV infection among children under 5 years old, by country. **(A,C,E)** ASIR in 2021. **(B,D,F)** AAPC from 1990 to 2021.

In 2021, the ASPR of HIV infection among WCBA was highest in Southern sub-Saharan Africa, followed closely by Eastern sub-Saharan Africa (Figure 2A). Globally, the ASPR of HIV infection among WCBA showed a significant upward trend from 1990 to 2021, except in Central Sub-Saharan Africa, where no significant increase was observed. The most pronounced rises occurred in Oceania (AAPC = 17.29%), Eastern Europe (AAPC = 12.84%), and South Asia (AAPC = 12.10%) (Supplementary file 1; Figure 2B). Similarly, Southern and Eastern Sub-Saharan Africa had the highest ASIR of HIV infection among children under five in 2021 (Figure 3A). The ASIR of HIV infection in this pediatric population also exhibited considerable regional heterogeneity. The most substantial increases were observed in Eastern Europe (AAPC = 10.30%), South Asia (AAPC = 10.22%), and Oceania (AAPC = 9.73%), whereas Australasia experienced the greatest decline (AAPC = −7.62%) (Supplementary file 2; Figure 3B).

In 2021, Central, Southern, and Eastern Sub-Saharan Africa had the highest ASPR of syphilis among WCBA and the highest ASIR among children under five across all 21 regions (Figures 2C, 3C). Notably, Southern Sub-Saharan Africa demonstrated the most substantial decline in ASPR from 1990 to 2021 (AAPC = −2.33%), while Tropical Latin America exhibited the greatest increase (AAPC = 1.26%) (Supplementary file 1; Figure 2D). From 1990 to 2021, Southern Asia experienced the most significant increase in ASIR of syphilis among children under five (AAPC = 1.05%), while Andean Latin America showed the most substantial decline (AAPC = −2.04%) (Supplementary file 2; Figure 3D).

The global burden of HBV infection among WCBA also showed distinct geographical patterns in 2021. While East and South Asia had the highest number of cases, Central and Western Sub-Saharan Africa bore the most severe disease burden, demonstrating the highest ASPR (Figure 2E). A consistent downward trend in HBV ASPR was observed across all GBD regions from 1990 to 2021. The most significant declines occurred in Central Europe (AAPC = −2.46%), East Asia (AAPC = −2.13%), and Central Latin America (AAPC = −2.13%) (Supplementary file 1; Figure 2F). Similar to the patterns for syphilis, Southern, Eastern, and Central Sub-Saharan Africa had the highest ASIR of HBV infection among children under five between 1990 and 2021 (Figure 3E). Furthermore, East Asia (AAPC = −9.36%) showed the most remarkable decrease in ASIR in this pediatric group, followed by Eastern Europe (AAPC = −6.97%) (Supplementary file 2; Figure 3F).

### 3.3 Age differences

We further analyzed the ASPR of three MTCT diseases among WCBA across different age groups (Supplementary file 3). For HIV infection, both ASPR and AAPC increased with age in groups below 40–44 years, with a slight decline observed in the 45–49-year age group. The 40–44-year group exhibited the highest ASPR (1,215.72 per 100,000 population) and the largest increase (AAPC = 6.19%) among all seven groups. In contrast, the global syphilis ASPR among WCBA peaked at younger ages, with the highest value observed in the 25–29-year age group, followed by a gradual decline with increasing age. Most age groups maintained stable ASPR levels during 2019–2021. Regarding HBV infection, the highest ASPR was also found in the 25–29-year age group, after which its prevalence decreases progressively with age. All age groups showed a declining trend in HBV infection prevalence between 2019 and 2021, with the most pronounced decrease occurring in the 15–19-year age group.

### 3.4 Trends and proportions at different SDI levels

We further investigated the association between the SDI and the prevalence among WCBA and incidence among children under five for the three diseases globally. The results demonstrated significant inverse correlations between SDI and disease burden metrics, with all three diseases exhibiting decreasing trends in ASPR and ASIR as SDI increased.

**Figure 4:**
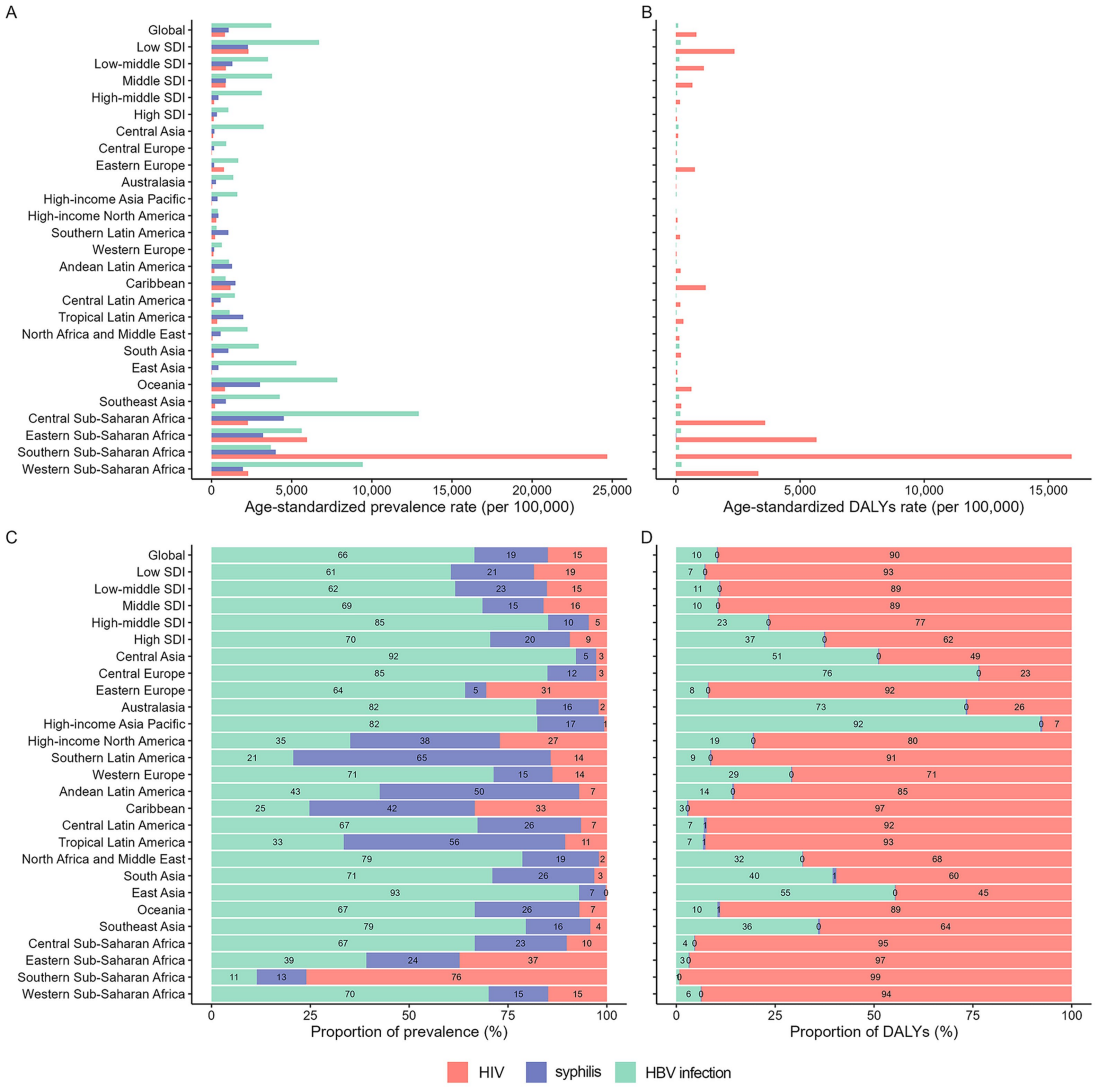
Proportion of prevalent cases and DALYs attributable to each infection among WCBA across 21 GBD regions, 2021. ASPR, age-standardized prevalence rate; DALYs, disability-adjusted life years.

**Figure 5:**
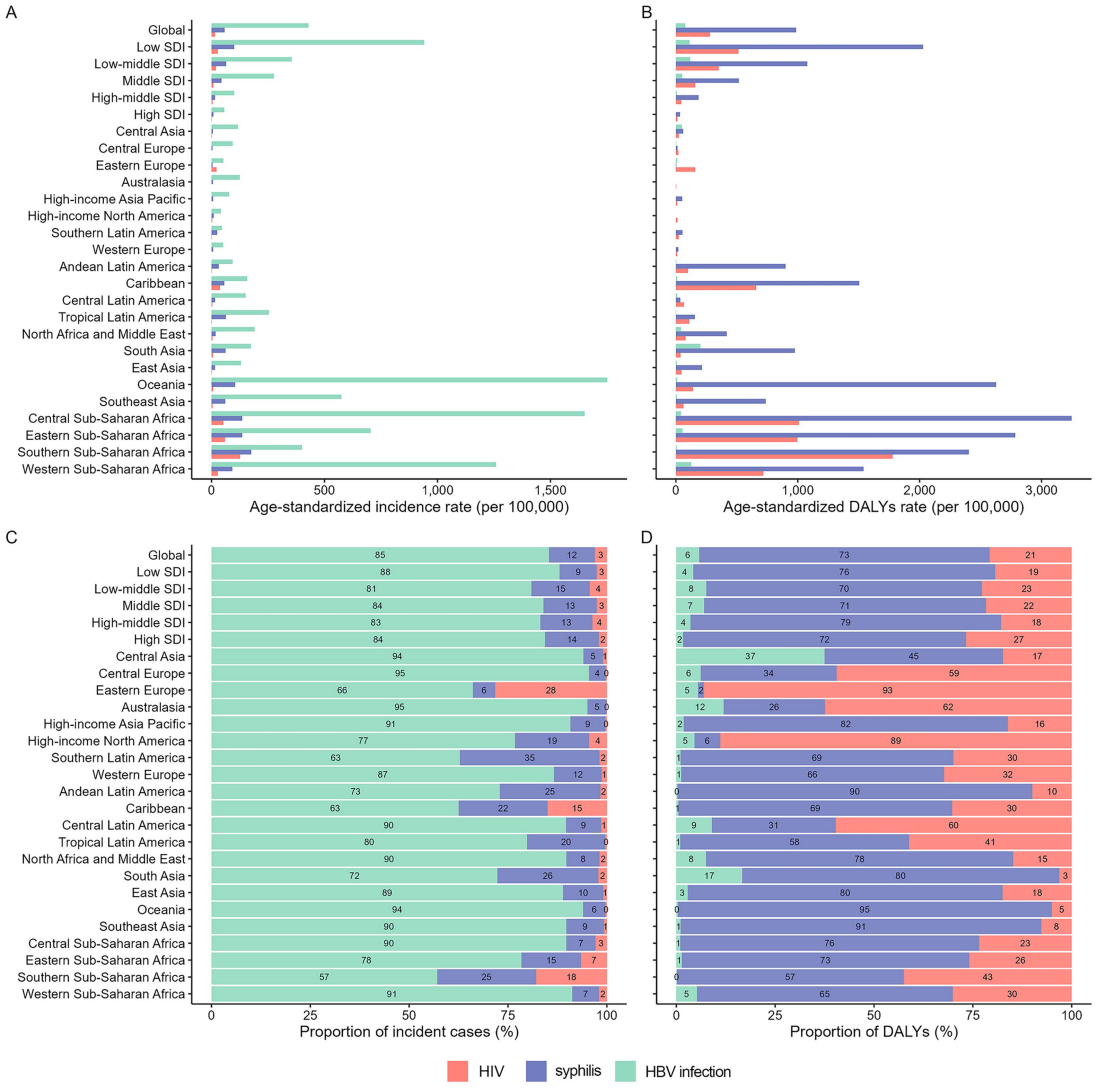
Proportion of new cases and DALYs attributable to each infection among children under 5 years old across 21 GBD regions, 2021. ASPR, age-standardized prevalence rate; DALYs, disability-adjusted life years.

Significant geographical heterogeneity was observed in the ASPR and DALYs among WCBA and ASIR and DALYs among children under 5 years old for the three MTCT diseases in 2021. Among WCBA, Southern sub-Saharan Africa had the highest HIV infection prevalence and DALYs rate, while Central sub-Saharan Africa showed the highest prevalence rates for both syphilis and HBV infection (Figures 4A,B). Among children under 5 years old, Oceania exhibited the highest HBV infection incidence, while Southern sub-Saharan Africa showed the highest incidence and DALYs rates for HIV infection (Figures 5A,B).

In 2021, HBV showed the highest prevalence among WCBA, whereas HIV infection imposed the greatest DALYs burden across most regions (Figures 4C,D). For children under five, HBV infection dominated the number of incident cases, while syphilis caused the most substantial DALYs burden (Figures 5C,D).

### 3.5 Prediction of global prevalence and DALYs among WCBA

Using the BAPC model, we predicted the ASPR and age-standardized DALY rate in the next 10 years. Our projections indicate a decline in the ASPR for both HIV and HBV infections from 2022 to 2030, whereas the prevalence of syphilis is expected to increase. The age-standardized DALY rate for HIV infection is projected to rise across all age groups. In contrast, the rates of syphilis and HBV infection are projected to remain relatively stable or show a slight decrease during the same period (Supplementary file 4).

## 4 Discussion

Since the onset of the COVID-19 pandemic, a significant resurgence in STIs has been observed globally, particularly among young adults, including those in high-income countries. This study provides a comprehensive estimation of the global prevalence among WCBA and incidence in children under five for three major MTCT infections—HIV, syphilis, and HBV infections—from 1990 to 2021, with predictions of prevalence trends among WCBA over the next decade. This targeted analysis delineates the global epidemiological landscape of these infections and offers evidence-based insights to inform public health policies and optimize intervention strategies at national and global levels.

### 4.1 Global overview

After three decades, the worldwide HIV response remains a significant health challenge, compounded by the ever-expanding population of people living with HIV (20). Consistent with previous studies, the ASPR of HIV among WCBA was increased globally from 1990 to 2021. HIV can be transmitted from infected mothers to their children during pregnancy, childbirth, or breastfeeding. More than 95% of pediatric HIV infections are attributable to MTCT. This transmission route carries substantial morbidity and mortality risks, particularly for neonates with immature immune systems, who are vulnerable to rapid disease progression and opportunistic infections (21). Owing to the implementation of enhanced prevention and control strategies worldwide, the ASIR of HIV among children under five decreased globally after 2002.

The prevalence of syphilis has been reported to correlate closely with HIV prevalence (22). This association may be due to factors that enhance the general transmission of STIs. Although syphilis can cause severe complications across all age groups, contemporary treatment strategies typically enable successful management of young adults. Overall, the disease burden of syphilis decreased before 2010, which may be attributed to improved access to antimicrobial treatment. Our findings are consistent with those of a previous study by Tao Chen et al. (23). Antibiotics are the most effective treatment for syphilis. However, with the widespread use of antimicrobial agents, antimicrobial resistance has become a growing threat to syphilis control (24). Furthermore, young people with multiple and occasional sexual partners, including men who have sex with men (MSMs), are at particularly high risk (25), which increases the likelihood of infection among WCBA. Untreated maternal infection can lead to adverse pregnancy outcomes, including congenital syphilis, which remains a major contributor to global morbidity and mortality. In developing countries, maternal syphilis infection contributes to thousands of stillbirths and neonatal deaths each year (26). Consistent with the disease burden observed among WCBA, the global burden of syphilis in children under five has also increased in recent years. Global health surveillance data also indicate a marked rise in the incidence of syphilis in children (27). Strengthening maternal and neonatal health services, improving maternal education, expanding prenatal interventions, and enhancing monitoring systems for affected youth are critical strategies to reduce the burden of syphilis (28).

HBV infection is the sixth leading cause of death worldwide and continues to pose a substantial global burden, affecting an estimated 296 million people. Of particular concern is that this population includes more than 6 million children under five (29). Globally, the disease burden of HBV infection among WCBA and 0–5-year-old children has shown a downward trend over the past three decades. This decline is largely attributable to the extensive implementation of global preventive measures, such as enhanced birth dose vaccination, prenatal hepatitis B screening, and risk reduction services, as well as increased research on functional cures for HBV infection (30).

### 4.2 Regional differences

Sub-Saharan Africa bears a disproportionate burden of the global HIV epidemic, accounting for approximately 67% of all people living with HIV worldwide (31). In this study, the highest prevalence of HIV among WCBA and children under five was also observed in regions of sub-Saharan Africa (including Central, Eastern, Southern, and Western), indicating that the HIV burden in these regions remains the most severe. However, these regions also demonstrated the most pronounced decline in DALY rate, likely attributable to concerted governmental efforts to mitigate the epidemic. Notably, the 2013 Ministerial Commitment on Comprehensive Sexuality Education and Sexual and Reproductive Health and Rights in Eastern and Southern Africa represents one of the most robust policy initiatives aimed at reducing new HIV infections among young women by addressing early and unintended pregnancies (32). It should be noted that the burden of HIV has increased significantly in Eastern Europe. According to UNAIDS, only 62% of people living with HIV in this region were aware of their status, and 51% received HIV treatment, resulting in an overall viral suppression rate of 48% (33). The region has implemented a comprehensive strategy encompassing enhanced preventive measures, widespread legal advocacy, targeted community education for high-risk populations, and efforts to reduce social discrimination, collectively contributing to controlling the HIV disease burden (34).

The geographical pattern of syphilis among women aged 15–49 years and children under five in our study was consistent with previous findings, showing that sub-Saharan Africa, Latin America, and Oceania had the highest burden (35). The increased prevalence of syphilis among pregnant women in East and Southeast Asia and sub-Saharan Africa from 2000 to 2020 may be related to the growing proportion of the sexually active population aged 15–64 years, particularly in sub-Saharan Africa (36). For children, the incidence of congenital syphilis rose significantly between 2015 and 2021. Potential contributing factors include a parallel increase in primary and secondary syphilis cases among WCBA, insufficient antenatal screening and appropriate treatment, and possible reinfection during pregnancy (37).

The ASPR among WCBA and ASIR among children under five decreased with regional heterogeneity. Globally, an estimated 4.5 million women with chronic HBV infection give birth each year, with the highest burden concentrated in Africa and the Western Pacific regions (30). Among GBD regions, East Asia had the largest decrease in prevalence among WCBA and incidence among children under five between 1990 and 2021. Universal infant immunization provided at no cost may be a key contributor to reducing the prevalence of chronic HBV infection in this region. China achieved a remarkable public health success by reducing the prevalence of chronic HBV infection by 90%, attributable to comprehensive healthcare system reforms supported by strong political and financial commitments (38). Western and Central sub-Saharan Africa had the largest disease burden from HBV infection worldwide in 2021. This may be due to poor postnatal coverage of hepatitis B vaccines, including low rates of timely birth administration for infants, ineffective screening programs in the general population, and lower disease awareness compared to other regions (39).

### 4.3 SDI differences

Our findings indicate a significant inverse correlation between the SDI and the overall disease burden of HIV, syphilis, and HBV infection. These inverse correlations suggest that socioeconomic development plays a crucial role in reducing the burden of MTCT diseases. The disparities in HIV burden are multifactorial, with low-SDI regions exhibiting a higher prevalence of complex socioeconomic and structural challenges—including poverty, systemic inequities, HIV-related stigma, and barriers to healthcare access—compared to high-SDI regions (40). In some low SDI and middle-low SDI regions, the observed increase in syphilis prevalence may reflect improved diagnostic capabilities, leading to increased case detection and reporting (41). Our study found that the substantial disease burden of HIV, syphilis, and HBV infections remains high in low SDI and middle-low SDI regions. Therefore, comprehensive strategies for effective control and management, including screening, health education, and syndromic management, should be implemented in these regions.

### 4.4 Future perspectives

Achieving the World Health Organization (WHO) targets for eliminating MTCT of HIV, syphilis, and hepatitis B remains a major public health challenge across all regions. Our study projects a decline in the ASPR of both HIV and HBV infections between 2022 and 2030, whereas the prevalence of syphilis is anticipated to increase during the same period. Reducing infection prevalence among WCBA is a powerful dual-benefit strategy that not only safeguards women’s own health but also serves as primary prevention for children, thereby breaking the cycle of intergenerational transmission. These infections share several characteristics that support an integrated public health approach: they can be readily diagnosed, and their MTCT can be effectively prevented through similar interventions.

The WHO advocates a coordinated “triple elimination” initiative to simultaneously eliminate MTCT of HIV, syphilis, and hepatitis B. The cornerstone of this strategy is to screen all pregnant women for HIV, syphilis, and hepatitis B surface antigen as early as possible during pregnancy (30). In recent years, a growing number of countries have implemented national campaigns aligned with this initiative. Significant progress has been observed in several regions, with 15 countries and territories having received WHO validation for eliminating MTCT of syphilis and/or HIV. Nevertheless, sustaining and expanding these achievements requires strengthened political commitment, robust policy frameworks, and comprehensive prevention, screening, and treatment programs. These efforts are particularly critical in lower SDI regions, where health systems face greater challenges in reducing new infections and mortality among women and children and achieving complete MTCT elimination (8).

### 4.5 Limitations

This study has several limitations. First, the primary data were obtained from the GBD study 2021. The GBD study 2021 estimates rely more heavily on data from economically developed countries, and the accuracy of raw data in some low- and middle-income countries may be limited, which is an inherent constraint of the dataset. Second, diagnostic criteria and detection methods vary across regions and over time. Surveillance and data collection systems may be less robust or incomplete for some or all of these infections among WCBA and infants. It should be noted that while many middle- and lower-income countries now have relatively robust HIV surveillance systems, HBV testing and surveillance are generally weaker, and syphilis detection and reporting capabilities are often substantially less developed.

## 5 Conclusion

This study utilized data from the GBD study 2021 to estimate the disease burdens of HIV, syphilis, and HBV infection among WCBA and children under five. The findings reveal distinct epidemiological trajectories for each infection. The ASPR of HIV among WCBA continues to increase, whereas the ASIR of HIV among children under five initially rose before declining. This pattern suggests that pediatric interventions have had a mitigating effect, though greater efforts targeting women are still urgently needed. Globally, both the ASPR of syphilis in WCBA and the ASIR among children under five decreased initially between 1990 and 2021, but have since rebounded, indicating a resurgence that warrants enhanced screening, treatment, and public health attention. In contrast, widespread vaccination and prevention efforts have contributed to a consistent decline in HBV infection burden among both populations. Nevertheless, current progress remains insufficient to meet the WHO 2030 viral hepatitis elimination targets, particularly in low- and middle-income SDI regions, where intensified efforts are critical.

## Data Availability

The original contributions presented in the study are included in the article/Supplementary material, further inquiries can be directed to the corresponding author.

## Footnotes

^1^ http://ghdx.healthdata.org/gbd-results-tool
