# Supplementary material for: Global burden of HIV, syphilis, and HBV infection among women of childbearing age and children under five: based on the Global Burden of Diseases Study 2021
Source: Front Public Health. 2025 Oct 16;13:1666408. doi: 10.3389/fpubh.2025.1666408 (PMC12571732; doi:10.3389/fpubh.2025.1666408)
Supplement: Supplementary file 1 [file Supplementary_file_1.docx]

Supplementary Material

**Supplementary Table 1.** ASPR of HIV, syphilis and HBV infection in WCBA of 1990 and 2021, and average annual percentage changes from 1990 to 2021

|  | Prevalence | | | | |
| --- | --- | --- | --- | --- | --- |
|  | 1990 | | 2021 | | AAPC in ASR, (1990-2021, 95%*CI*) |
|  | Number | ASR (per 100000 population) | Number | ASR (per 100000 population) |  |
| **HIV** |  |  |  |  |  |
| Global | 3554833 (3296708-3796481) | 260.91 (238.39-284.17) | 16497874 (15705722-17407602) | 830.87 (784.57-884.29) | 3.85 (3.48 to 4.22)^*^ |
| SDI |  |  |  |  |  |
| Low SDI | 2067982 (1856935-2293210) | 1853.22 (1619.77-2102.34) | 5569700 (5073342-6207982) | 2293.68 (2082.78-2561.02) | 0.74 (0.41 to 1.07)^*^ |
| Low-middle SDI | 1064387 (954339-1162514) | 381.70 (336.04-427.47) | 4356055 (4084547-4636336) | 889.85 (820.96-958.68) | 2.80 (2.31 to 3.30)^*^ |
| Middle SDI | 188954 (164971-215710) | 41.65 (36.03-47.79) | 5624969 (5303093-6006754) | 870.23 (812.73-934.69) | 10.29 (9.82 to 10.76)^*^ |
| High-middle SDI | 55850 (46978-65436) | 19.88 (16.43-23.71) | 565981 (427249-736784) | 167.59 (122.84-235.73) | 7.13 (7.00 to 7.26)^*^ |
| High SDI | 173790 (114548-250348) | 74.47 (48.10-107.99) | 369319 (217336-533735) | 136.83 (79.73-200.49) | 1.98 (1.88 to 2.08)^*^ |
| Region |  |  |  |  |  |
| East Asia | 10557 (6120-14341) | 3.27 (1.89-4.47) | 70329 (36248-136909) | 19.87 (9.93-38.50) | 6.00 (5.87 to 6.13)^*^ |
| South Asia | 9430 (6312-13359) | 3.71 (2.38-5.53) | 653152 (534455-844530) | 135.54 (106.28-182.77) | 12.10 (11.10 to 13.11)^*^ |
| Central Asia | 1277 (930-1709) | 7.48 (5.42-10.06) | 24259 (18691-34632) | 94.21 (65.92-145.95) | 8.51 (8.29 to 8.74)^*^ |
| Southeast Asia | 18793 (13057-25223) | 15.04 (10.25-20.74) | 417222 (309976-555268) | 222.44 (162.02-306.89) | 8.97 (8.78 to 9.16)^*^ |
| Eastern Europe | 10524 (7085-15679) | 18.26 (12.22-27.46) | 419095 (310504-555392) | 768.52 (530.68-1152.95) | 12.84 (12.51 to 13.16)^*^ |
| Western Europe | 58501 (46656-71052) | 60.07 (47.27-74.00) | 125399 (93255-156664) | 119.74 (88.29-151.47) | 2.25 (2.18 to 2.33)^*^ |
| Central Europe | 983 (622-1356) | 3.20 (1.94-4.53) | 8548 (5525-12416) | 31.18 (19.03-47.88) | 7.61 (7.31 to 7.92)^*^ |
| High-income Asia Pacific | 1008 (513-1641) | 2.21 (1.06-3.61) | 5811 (3686-8220) | 14.57 (8.95-20.94) | 6.25 (6.09 to 6.41)^*^ |
| Oceania | 91 (66-123) | 5.89 (4.17-8.06) | 27681 (21615-34078) | 834.27 (637.31-1054.97) | 17.29 (16.54 to 18.05)^*^ |
| Australasia | 606 (373-864) | 11.10 (6.51-16.08) | 2761 (1410-4525) | 35.81 (18.35-59.39) | 3.85 (3.78 to 3.91)^*^ |
| High-income North America | 142318 (87289-212651) | 182.40 (109.54-275.37) | 262743 (137654-403564) | 291.50 (149.54-455.16) | 1.53 (1.44 to 1.62)^*^ |
| Andean Latin America | 2554 (1676-3919) | 26.65 (17.35-40.42) | 31291 (22663-42248) | 177.56 (126.04-250.15) | 6.31 (6.19 to 6.43)^*^ |
| Central Latin America | 9958 (7152-13697) | 24.79 (17.68-34.53) | 97511 (66516-136657) | 141.84 (95.10-203.82) | 5.76 (5.68 to 5.83)^*^ |
| Tropical Latin America | 40513 (27402-58149) | 99.70 (67.32-143.45) | 224913 (134954-341730) | 351.47 (203.83-565.27) | 4.14 (4.02 to 4.25)^*^ |
| Southern Latin America | 9169 (7997-10299) | 74.65 (61.95-88.88) | 39943 (33736-47737) | 219.43 (182.79-263.14) | 3.53 (3.47 to 3.60)^*^ |
| Caribbean | 70735 (49329-93794) | 752.45 (464.22-1100.84) | 143514 (113220-170578) | 1176.61 (909.15-1455.55) | 1.46 (1.24 to 1.67)^*^ |
| Eastern Sub-Saharan Africa | 1893605 (1695299-2083600) | 4401.92 (3854.99-4976.96) | 5528688 (5048111-6193161) | 5956.09 (5386.15-6665.83) | 0.99 (0.80 to 1.18)^*^ |
| Southern Sub-Saharan Africa | 457909 (402428-514704) | 3357.73 (2788.30-3974.73) | 5331459 (5099953-5596957) | 24688.66 (23436.21-26058.78) | 6.64 (5.93 to 7.36)^*^ |
| Western Sub-Saharan Africa | 523285 (421402-629844) | 1221.05 (974.02-1510.39) | 2339303 (2132648-2562205) | 2279.67 (2056.35-2506.20) | 1.98 (1.79 to 2.17)^*^ |
| North Africa and Middle East | 4808 (2284-12489) | 6.31 (3.01-16.82) | 94445 (49528-198852) | 58.61 (30.85-122.70) | 7.42 (7.23 to 7.62)^*^ |
| Central Sub-Saharan Africa | 288209 (224833-360979) | 2374.96 (1801.73-3095.01) | 649806 (539184-767647) | 2254.97 (1865.53-2705.14) | -0.18 (-0.38 to 0.02) |
| **syphilis** |  |  |  |  |  |
| Global | 14038355 (10599589-18399106) | 1028.29 (653.67-1537.16) | 20475289 (15154285-27783875) | 1057.32 (646.18-1626.10) | 0.07 (-0.05 to 0.20) |
| SDI |  |  |  |  |  |
| Low SDI | 3769532 (2977121-4695511) | 3297.18 (2264.80-4650.15) | 6300025 (4603829-8399217) | 2269.35 (1394.14-3468.04) | -1.31 (-1.59 to -1.03)^*^ |
| Low-middle SDI | 4361104 (3236618-5802665) | 1580.20 (988.73-2387.51) | 6645643 (4894267-9049357) | 1298.86 (797.80-1994.48) | -0.67 (-0.77 to -0.57)^*^ |
| Middle SDI | 4124109 (3062904-5415506) | 909.71 (565.90-1382.87) | 5438130 (4019728-7281702) | 887.10 (541.73-1364.28) | -0.10 (-0.21 to 0.01) |
| High-middle SDI | 999491 (717057-1338143) | 354.62 (207.89-563.45) | 1278164 (923093-1694878) | 425.84 (251.64-673.39) | 0.54 (0.38 to 0.69)^*^ |
| High SDI | 771673 (552312-1045725) | 336.03 (195.23-542.16) | 795641 (569528-1069823) | 327.62 (189.62-530.02) | -0.12 (-0.30 to 0.07) |
| Region |  |  |  |  |  |
| East Asia | 1274356 (913622-1721842) | 383.94 (223.20-611.66) | 1399766 (988230-1897262) | 419.71 (243.82-672.43) | 0.25 (-0.43 to 0.93) |
| South Asia | 3623206 (2630329-4873818) | 1411.21 (866.97-2174.57) | 5254614 (3794409-7207149) | 1051.82 (639.13-1638.30) | -0.98 (-1.39 to -0.56)^*^ |
| Central Asia | 31550 (22268-42049) | 181.50 (105.77-285.78) | 45036 (32490-60165) | 182.84 (110.01-280.42) | 0.59 (0.04 to 1.15) |
| Southeast Asia | 1100324 (771261-1497643) | 888.54 (523.56-1415.56) | 1628559 (1179348-2156672) | 893.52 (537.74-1400.06) | 0.07 (-0.31 to 0.45) |
| Eastern Europe | 92013 (65173-126015) | 161.77 (93.12-260.44) | 72790 (52476-97878) | 150.27 (87.17-240.43) | -0.23 (-0.31 to -0.15)^*^ |
| Western Europe | 145139 (104191-192486) | 150.95 (87.85-243.65) | 136047 (96941-178663) | 150.08 (87.04-240.55) | -0.01 (-0.06 to 0.04) |
| Central Europe | 50475 (36113-67225) | 164.38 (94.04-265.79) | 38902 (28341-50501) | 152.43 (89.88-242.31) | -0.23 (-0.35 to -0.10)^*^ |
| High-income Asia Pacific | 180344 (130132-240948) | 400.55 (232.72-649.38) | 137769 (99122-183088) | 370.13 (213.16-602.59) | -0.24 (-0.27 to -0.21)^*^ |
| Oceania | 53187 (39583-70659) | 3385.71 (2087.58-5159.32) | 105537 (75966-141685) | 3014.50 (1810.21-4674.20) | -0.38 (-0.54 to -0.23)^*^ |
| Australasia | 16062 (11374-21328) | 297.31 (171.59-480.95) | 20292 (14369-27374) | 279.78 (159.59-455.47) | -0.19 (-0.21 to -0.18)^*^ |
| High-income North America | 355465 (252858-489844) | 466.75 (268.66-753.48) | 366342 (264666-500019) | 435.56 (251.29-704.77) | -0.22 (-0.33 to -0.12)^*^ |
| Andean Latin America | 138167 (95757-188782) | 1400.92 (826.19-2184.26) | 225384 (159125-313419) | 1272.85 (742.82-2031.91) | -0.35 (-0.52 to -0.19)^*^ |
| Central Latin America | 269034 (192450-364365) | 635.94 (369.21-1012.77) | 388527 (283911-526632) | 569.08 (333.64-901.71) | -0.23 (-0.42 to -0.04) |
| Tropical Latin America | 541365 (385993-725418) | 1337.08 (782.87-2112.65) | 1192268 (959706-1495887) | 1976.11 (1335.83-2800.75) | 1.26 (0.97 to 1.55)^*^ |
| Southern Latin America | 122603 (90747-158408) | 986.15 (595.95-1536.17) | 182700 (133785-246465) | 1050.48 (613.66-1664.52) | 0.59 (-0.15 to 1.34) |
| Caribbean | 133400 (99356-172654) | 1392.35 (890.00-2075.45) | 179861 (132301-237069) | 1492.53 (910.78-2286.52) | 0.73 (0.58 to 0.88)^*^ |
| Eastern Sub-Saharan Africa | 2384903 (1952007-2889492) | 5338.26 (3862.81-7275.05) | 3486184 (2558678-4595677) | 3201.58 (1990.43-4876.76) | -1.62 (-1.80 to -1.44)^*^ |
| Southern Sub-Saharan Africa | 1105847 (880626-1357959) | 8110.28 (5685.39-11170.64) | 881218 (642500-1182779) | 3997.33 (2416.17-6192.65) | -2.33 (-2.60 to -2.06)^*^ |
| Western Sub-Saharan Africa | 1240200 (928220-1616952) | 2797.94 (1776.35-4144.86) | 2370058 (1701680-3158937) | 1954.44 (1181.00-3004.97) | -1.15 (-1.24 to -1.06)^*^ |
| North Africa and Middle East | 474359 (332028-643173) | 608.56 (350.83-966.71) | 885582 (618006-1173990) | 555.31 (322.83-872.67) | -0.35 (-0.68 to -0.01) |
| Central Sub-Saharan Africa | 706358 (536153-925398) | 5667.40 (3617.15-8402.25) | 1477853 (1074382-1985235) | 4496.42 (2721.25-6956.89) | -0.94 (-1.12 to -0.76)^*^ |
| **HBV infection** |  |  |  |  |  |
| Global | 74148652 (66546143-82837534) | 5505.12 (4917.72-6184.61) | 73346811 (66461387-81181136) | 3735.41 (3339.92-4172.06) | -1.25 (-1.28 to -1.22)^*^ |
| SDI |  |  |  |  |  |
| Low SDI | 9389812 (8479518-10452719) | 8315.24 (7449.14-9247.04) | 18225607 (16625539-20136848) | 6702.57 (6064.61-7433.13) | -0.69 (-0.74 to -0.63)^*^ |
| Low-middle SDI | 12917987 (11534231-14461338) | 4712.64 (4196.06-5304.60) | 17685873 (15994360-19622738) | 3518.87 (3158.92-3930.97) | -0.92 (-0.97 to -0.87)^*^ |
| Middle SDI | 31480514 (28098534-35321303) | 7036.93 (6252.21-7965.49) | 24078199 (21589324-26780573) | 3778.66 (3340.91-4247.48) | -2.02 (-2.07 to -1.97)^*^ |
| High-middle SDI | 16084404 (14417614-17887030) | 5762.71 (5133.32-6498.28) | 10536369 (9296430-11877675) | 3123.16 (2737.95-3541.93) | -1.99 (-2.19 to -1.80)^*^ |
| High SDI | 4234534 (3881128-4629726) | 1865.17 (1691.63-2065.22) | 2781141 (2538144-3052958) | 1048.33 (944.88-1164.18) | -1.85 (-1.97 to -1.73)^*^ |
| Region |  |  |  |  |  |
| East Asia | 33867975 (30346511-37841808) | 10170.24 (9057.78-11498.61) | 19345307 (16959037-21746551) | 5295.58 (4626.36-5992.09) | -2.13 (-2.31 to -1.95)^*^ |
| South Asia | 9442526 (8357655-10672233) | 3689.71 (3238.93-4196.80) | 14499034 (12969212-16217609) | 2934.25 (2605.33-3304.76) | -0.72 (-0.75 to -0.70)^*^ |
| Central Asia | 664458 (555797-761898) | 3925.51 (3282.94-4525.32) | 813455 (686362-921715) | 3248.79 (2715.23-3701.93) | -0.62 (-0.65 to -0.59)^*^ |
| Southeast Asia | 7973081 (7208321-8836185) | 6609.15 (5927.17-7385.05) | 7926275 (7181756-8756657) | 4254.17 (3813.41-4742.16) | -1.40 (-1.46 to -1.34)^*^ |
| Eastern Europe | 1466688 (1289751-1664500) | 2651.36 (2295.73-3041.21) | 879771 (778558-992212) | 1660.48 (1452.56-1890.37) | -1.49 (-1.64 to -1.33)^*^ |
| Western Europe | 1061348 (944577-1204492) | 1109.50 (972.73-1268.93) | 650405 (580251-734796) | 643.03 (564.82-733.78) | -1.74 (-1.83 to -1.65)^*^ |
| Central Europe | 601000 (547561-657056) | 1956.04 (1749.83-2170.79) | 269048 (243634-297496) | 906.66 (811.00-1016.94) | -2.46 (-2.59 to -2.32)^*^ |
| High-income Asia Pacific | 1328626 (1200098-1457208) | 2913.08 (2597.40-3224.16) | 670555 (614238-734413) | 1597.96; (1441.73-1785.22) | -1.93 (-2.07 to -1.78)^*^ |
| Oceania | 178666 (160816-197709) | 11457.35 (10187.19-12791.56) | 265445 (237272-293398) | 7842.70 (6962.41-8759.40) | -1.22 (-1.27 to -1.18)^*^ |
| Australasia | 123659 (110831-137647) | 2301.21 (2025.11-2593.35) | 106253 (95146-118578) | 1355.38 (1199.52-1536.13) | -1.73 (-1.84 to -1.61)^*^ |
| High-income North America | 464237 (416100-528926) | 621.48 (547.86-712.88) | 340158 (306807-379153) | 384.65 (343.58-430.51) | -1.47 (-1.58 to -1.36)^*^ |
| Andean Latin America | 130771 (114228-150791) | 1401.46 (1206.76-1638.64) | 189745 (166316-219011) | 1078.78 (934.95-1261.32) | -0.84 (-0.88 to -0.80)^*^ |
| Central Latin America | 1185315 (991579-1382860) | 2810.35 (2346.09-3307.52) | 1001227 (851686-1148969) | 1455.36 (1236.04-1688.80) | -2.13 (-2.26 to -2.00)^*^ |
| Tropical Latin America | 826000 (719618-956516) | 2070.67 (1781.62-2417.35) | 713230 (614235-829336) | 1115.85 (956.72-1304.63) | -2.01 (-2.12 to -1.90)^*^ |
| Southern Latin America | 51730 (43363-61138) | 418.85 (347.82-499.86) | 58057 (49719-68879) | 321.84 (272.78-385.65) | -0.86 (-1.04 to -0.68)^*^ |
| Caribbean | 108215 (90965-126355) | 1153.29 (963.07-1352.87) | 106337 (91669-122796) | 879.47 (750.09-1025.74) | -0.85 (-1.02 to -0.68)^*^ |
| Eastern Sub-Saharan Africa | 3321259 (2976583-3694752) | 7660.12 (6783.31-8572.85) | 5813197 (5228712-6434182) | 5621.67 (5011.94-6287.53) | -0.98 (-1.05 to -0.91)^*^ |
| Southern Sub-Saharan Africa | 764131 (676295-862099) | 5698.49 (4994.73-6466.03) | 801576 (711543-899737) | 3682.51 (3226.17-4171.85) | -1.42 (-1.49 to -1.36)^*^ |
| Western Sub-Saharan Africa | 5459323 (4943271-6063068) | 12459.11 (11220.72-13821.04) | 11048481 (9997603-12246050) | 9426.98 (8506.35-10491.01) | -0.88 (-0.92 to -0.85)^*^ |
| North Africa and Middle East | 3208565 (2945366-3512274) | 4119.23 (3749.64-4529.02) | 3605894 (3348110-3892953) | 2239.57 (2054.86-2439.82) | -1.95 (-2.03 to -1.88)^*^ |
| Central Sub-Saharan Africa | 1921077 (1737734-2117972) | 15340.27 (13703.77-16982.49) | 4243361 (3870339-4711208) | 12922.87 (11681.69-14400.69) | -0.54 (-0.59 to -0.50)^*^ |

**Supplementary Table 2.** ASIR of HIV, syphilis and HBV infection in children under 5 years old of 1990 and 2021, and average annual percentage changes from 1990 to 2021

|  | Incidence | | | | |
| --- | --- | --- | --- | --- | --- |
|  | 1990 | | 2021 | | AAPC in ASR, (1990-2021, 95%*CI*) |
|  | Count | ASR (per 100000 population) | Count | ASR (per 100000 population) |  |
| **HIV** |  |  |  |  |  |
| Global | 256870 (232182-280577) | 41.43 (37.45-45.26) | 101274 (84106-126532) | 15.39 (12.78-19.22) | -3.23  (-3.94 to -2.50)^*^ |
| SDI |  |  |  |  |  |
| Low SDI | 160586 (140220-180629) | 176.87 (154.44-198.94) | 45769 (31256-68484) | 27.64 (18.88-41.36) | -5.92 (-6.82 to -5.02)^*^ |
| Low-middle SDI | 84467 (74665-93578) | 48.69 (43.04-53.94) | 36745 (29257-46014) | 19.18 (15.27-24.02) | -3.04 (-3.76 to -2.32)^*^ |
| Middle SDI | 7404 (6190-8600) | 3.69 (3.09-4.29) | 14860 (12401-18608) | 8.41 (7.02-10.54) | 2.20 (0.63 to 3.79) |
| High-middle SDI | 1076 (859-1301) | 1.16 (0.92-1.40) | 3091 (2076-4821) | 4.41 (2.96-6.88) | 4.57 (2.45 to 6.74)^*^ |
| High SDI | 3057 (1913-4444) | 4.95 (3.10-7.20) | 713 (430-1015) | 1.32 (0.80-1.89) | -4.65 (-5.24 to -4.05)^*^ |
| Region |  |  |  |  |  |
| East Asia | 224 (108-361) | 0.19 (0.09-0.31) | 1094 (516-2222) | 1.37 (0.64-2.77) | 6.47 (5.40 to 7.55)^*^ |
| South Asia | 392 (240-608) | 0.25 (0.15-0.39) | 8326 (5381-14967) | 5.25 (3.39-9.44) | 10.22 (9.76 to 10.67)^*^ |
| Central Asia | 83 (53-123) | 0.87 (0.56-1.29) | 128 (77-281) | 1.28 (0.77-2.81) | 1.41 (-0.85 to 3.72) |
| Southeast Asia | 636 (435-860) | 1.09 (0.75-1.48) | 2795 (1569-5004) | 4.97 (2.79-8.89) | 4.59 (3.42 to 5.77)^*^ |
| Eastern Europe | 191 (111-318) | 1.11 (0.65-1.85) | 2255 (1613-3584) | 22.28 (15.94-35.42) | 10.30 (8.90 to 11.71)^*^ |
| Western Europe | 1002 (747-1282) | 4.36 (3.25-5.59) | 164 (81-269) | 0.77 (0.38-1.27) | -5.51 (-9.20 to -1.67)^*^ |
| Central Europe | 20 (11-30) | 0.21 (0.12-0.33) | 16 (10-25) | 0.29 (0.18-0.45) | 1.04 (-0.82 to 2.95) |
| High-income Asia Pacific | 12 (6-20) | 0.12 (0.06-0.20) | 21 (11-34) | 0.33 (0.16-0.53) | 3.45 (1.15 to 5.80)^*^ |
| Oceania | 4 (3-7) | 0.43 (0.27-0.65) | 142 (115-169) | 7.36 (5.96-8.74) | 9.72 (7.93 to 11.53)^*^ |
| Australasia | 16 (9-23) | 1.01 (0.57-1.51) | 2 (0-6) | 0.10 (0.02-0.32) | -7.62 (-11.35 to -3.75)^*^ |
| High-income North America | 2409 (1386-3671) | 11.11 (6.39-16.93) | 498 (255-774) | 2.43 (1.25-3.78) | -5.27 (-5.88 to -4.65)^*^ |
| Andean Latin America | 108 (61-184) | 2.05 (1.15-3.48) | 131 (36-293) | 2.13 (0.58-4.75) | 0.01 (-1.37 to 1.40) |
| Central Latin America | 364 (238-539) | 1.58 (1.03-2.34) | 505 (167-1068) | 2.51 (0.83-5.32) | 1.54 (0.19 to 2.91)^*^ |
| Tropical Latin America | 1047 (636-1628) | 6.13 (3.72-9.53) | 241 (135-450) | 1.40 (0.78-2.62) | -4.70 (-5.21 to -4.18)^*^ |
| Southern Latin America | 219 (181-259) | 4.26 (3.52-5.04) | 57 (25-139) | 1.34 (0.57-3.24) | -4.02 (-5.53 to -2.49)^*^ |
| Caribbean | 4924 (3287-6659) | 119.19 (79.56-161.19) | 1463 (365-2815) | 37.83 (9.44-72.77) | -3.62 (-5.00 to -2.22)^*^ |
| Eastern Sub-Saharan Africa | 153212 (134355-170936) | 424.57 (372.32-473.68) | 37430 (23734-59170) | 58.67 (37.20-92.75) | -6.32 (-7.40 to -5.24)^*^ |
| Southern Sub-Saharan Africa | 30647 (26296-35090) | 410.11 (351.88-469.56) | 10118 (8472-11516) | 126.01 (105.51-143.43) | -4.14 (-5.47 to -2.78)^*^ |
| Western Sub-Saharan Africa | 40346 (31592-49535) | 112.87 (88.38-138.58) | 22340 (18934-26527) | 27.94 (23.68-33.18) | -4.60 (-5.72 to -3.46)^*^ |
| North Africa and Middle East | 334 (122-998) | 0.65 (0.24-1.95) | 2417 (805-6349) | 3.95 (1.32-10.39) | 5.85 (5.43 to 6.27)^*^ |
| Central Sub-Saharan Africa | 20681 (15545-27120) | 199.15 (149.69-261.16) | 11129 (5520-18246) | 52.83 (26.20-86.61) | -4.47 (-5.22 to -3.71)^*^ |
| **syphilis** |  |  |  |  |  |
| Global | 383848 (301578-483750) | 61.92 (48.65-78.03) | 384121 (314943-460834) | 58.36 (47.85-70.02) | -0.10 (-0.44 to 0.25)^*^ |
| SDI |  |  |  |  |  |
| Low SDI | 142808 (124751-165993) | 157.28 (137.40-182.82) | 166652 (147362-187990) | 100.65 (89.00-113.54) | -1.44 (-1.65 to -1.24)^*^ |
| Low-middle SDI | 136066 (104438-174848) | 78.43 (60.20-100.79) | 123302 (98354-151321) | 64.36 (51.34-78.99) | -0.64 (-0.99 to -0.29)^*^ |
| Middle SDI | 83615 (57232-115456) | 41.70 (28.54-57.57) | 77878 (56862-101574) | 44.09 (32.19-57.51) | 0.19 (-0.10 to 0.49) |
| High-middle SDI | 14846 (10779-19781) | 15.98 (11.60-21.29) | 11081 (8533-14167) | 15.82 (12.18-20.23) | -0.03 (-0.24 to 0.18) |
| High SDI | 6232 (4195-8847) | 10.10 (6.80-14.34) | 4904 (3525-6527) | 9.11 (6.55-12.12) | -0.32 (-0.49 to -0.15)^*^ |
| Region |  |  |  |  |  |
| East Asia | 22410 (14578-31880) | 19.36 (12.59-27.54) | 11891 (8510-15934) | 14.85 (10.63-19.90) | -0.82 (-1.73 to 0.10) |
| South Asia | 115701 (80554-158924) | 73.68 (51.30-101.21) | 97512 (70904-127622) | 61.49 (44.71-80.47) | -0.54 (-1.11 to 0.03) |
| Central Asia | 549 (495-611) | 5.77 (5.20-6.41) | 604 (561-655) | 6.04 (5.61-6.55) | 0.13 (0.02 to 0.23)^*^ |
| Southeast Asia | 25489 (17838-35029) | 43.73 (30.60-60.09) | 34180 (24837-44651) | 60.73 (44.13-79.33) | 1.05 (0.87 to 1.22)^*^ |
| Eastern Europe | 660 (400-1010) | 3.83 (2.32-5.86) | 446 (292-645) | 4.41 (2.89-6.37) | 0.46 (0.10 to 0.81)^*^ |
| Western Europe | 1359 (868-2070) | 5.92 (3.78-9.02) | 1507 (1079-2045) | 7.10 (5.08-9.63) | 0.61 (0.18 to 1.04)^*^ |
| Central Europe | 361 (282-460) | 3.95 (3.09-5.04) | 235 (183-298) | 4.21 (3.27-5.34) | 0.19 (-0.08 to 0.46) |
| High-income Asia Pacific | 1040 (794-1361) | 10.18 (7.77-13.32) | 490 (369-642) | 7.60 (5.72-9.95) | -0.97 (-1.15 to -0.79)^*^ |
| Oceania | 1340 (1270-1416) | 133.44 (126.49-141.02) | 2025 (1945-2106) | 104.66 (100.53-108.87) | -0.85 (-1.17 to -0.54)^*^ |
| Australasia | 116 (100-134) | 7.54 (6.47-8.72) | 115 (102-128) | 6.31 (5.59-7.04) | -0.63 (-0.91 to -0.35)^*^ |
| High-income North America | 2733 (1703-4116) | 12.60 (7.85-18.98) | 2071 (1429-2870) | 10.10 (6.97-14.00) | -0.70 (-1.04 to -0.36)^*^ |
| Andean Latin America | 3191 (3084-3306) | 60.41 (58.39-62.59) | 1998 (1926-2071) | 32.45 (31.29-33.65) | -2.04 (-2.33 to -1.75)^*^ |
| Central Latin America | 4638 (3766-5706) | 20.15 (16.36-24.79) | 2989 (2632-3411) | 14.88 (13.10-16.98) | -1.01 (-1.21 to -0.81)^*^ |
| Tropical Latin America | 7959 (5169-11377) | 46.60 (30.26-66.61) | 10795 (7613-14745) | 62.74 (44.24-85.69) | 0.95 (0.74 to 1.15)^*^ |
| Southern Latin America | 1324 (1272-1374) | 25.72 (24.71-26.70) | 1080 (1042-1121) | 25.24 (24.36-26.20) | -0.10 (-0.69 to 0.50) |
| Caribbean | 2391 (2286-2505) | 57.86 (55.33-60.63) | 2186 (2110-2265) | 56.52 (54.54-58.55) | -0.02 (-0.16 to 0.12) |
| Eastern Sub-Saharan Africa | 88164 (75572-103999) | 244.31 (209.42-288.19) | 86364 (77311-96684) | 135.37 (121.18-151.55) | -1.88 (-2.05 to -1.71)^*^ |
| Southern Sub-Saharan Africa | 21094 (15121-28383) | 282.27 (202.35-379.81) | 14088 (10669-17957) | 175.46 (132.88-223.65) | -1.52 (-1.76 to -1.29)^*^ |
| Western Sub-Saharan Africa | 45258 (38362-54227) | 126.62 (107.32-151.71) | 74014 (61856-87805) | 92.57 (77.36-109.81) | -0.98 (-1.12 to -0.84)^*^ |
| North Africa and Middle East | 12647 (12096-13342) | 24.69 (23.61-26.04) | 10835 (10427-11327) | 17.72 (17.05-18.53) | -1.11 (-1.32 to -0.89)^*^ |
| Central Sub-Saharan Africa | 25425 (25035-25796) | 244.84 (241.08-248.41) | 28697 (28332-29087) | 136.22 (134.49-138.07) | -1.92 (-2.07 to -1.76)^*^ |
| **HBV infection** |  |  |  |  |  |
| Global | 9815411 (6555008-13255783) | 1583.29 (1057.37-2138.24) | 2827488 (1934294-3779226) | 429.60 (293.89-574.20) | -4.14 (-4.47 to -3.81)^*^ |
| SDI |  |  |  |  |  |
| Low SDI | 2157908 (1450166-2853640) | 2376.67 (1597.18-3142.93) | 1559072 (1076424-2078018) | 941.61 (650.11-1255.03) | -2.93 (-3.14 to -2.72)^*^ |
| Low-middle SDI | 2432477 (1576139-3305885) | 1402.14 (908.53-1905.60) | 679117 (452723-921668) | 354.49 (236.31-481.09) | -4.29 (-4.69 to -3.89)^*^ |
| Middle SDI | 3526723 (2381168-4768601) | 1758.65 (1187.40-2377.93) | 486397 (323341-656532) | 275.40 (183.07-371.73) | -5.90 (-6.14 to -5.65)^*^ |
| High-middle SDI | 1418011 (958604-1876330) | 1526.34 (1031.84-2019.67) | 69917 (46326-95539) | 99.82 (66.14-136.40) | -8.48 (-8.90 to -8.06)^*^ |
| High SDI | 274647 (194635-359274) | 445.05 (315.39-582.18) | 30209 (21622-38790) | 56.10 (40.16-72.04) | -6.37 (-6.88 to -5.85)^*^ |
| Region |  |  |  |  |  |
| East Asia | 2958434 (2023753-3969425) | 2555.91 (1748.40-3429.34) | 104119 (68267-144642) | 130.03 (85.26-180.64) | -9.36 (-9.58 to -9.13)^*^ |
| South Asia | 1647867 (1041598-2287124) | 1049.42 (663.33-1456.53) | 276449 (184114-375625) | 174.31 (116.09-236.85) | -5.46 (-5.80 to -5.12)^*^ |
| Central Asia | 73165 (56807-91171) | 768.17 (596.43-957.23) | 11627 (9109-14817) | 116.31 (91.12-148.21) | -5.93 (-6.57 to -5.28)^*^ |
| Southeast Asia | 1168789 (785777-1580815) | 2005.14 (1348.06-2712.00) | 323641 (215038-437045) | 575.01 (382.05-776.49) | -4.11 (-4.46 to -3.75)^*^ |
| Eastern Europe | 86660 (54065-123360) | 502.61 (313.56-715.46) | 5274 (3411-7399) | 52.12 (33.71-73.12) | -6.97 (-7.53 to -6.42)^*^ |
| Western Europe | 87962 (62170-114243) | 383.16 (270.82-497.65) | 10842 (7557-13988) | 51.07 (35.60-65.89) | -6.28 (-6.51 to -6.04)^*^ |
| Central Europe | 57380 (39109-76440) | 628.38 (428.29-837.10) | 5253 (3582-7124) | 94.04 (64.13-127.54) | -6.06 (-7.30 to -4.81)^*^ |
| High-income Asia Pacific | 62018 (46640-79294) | 607.07 (456.54-776.18) | 5013 (3749-6407) | 77.70 (58.11-99.30) | -5.86 (-6.87 to -4.83)^*^ |
| Oceania | 22607 (15166-31279) | 2251.29 (1510.31-3114.87) | 33886 (23282-45505) | 1751.69 (1203.55-2352.34) | -0.81 (-0.96 to -0.67)^*^ |
| Australasia | 14590 (9596-19781) | 946.00 (622.21-1282.61) | 2258 (1498-3030) | 124.35 (82.51-166.85) | -6.50 (-6.95 to -6.04)^*^ |
| High-income North America | 55578 (38391-71628) | 256.33 (177.07-330.36) | 8497 (5978-10809) | 41.45 (29.16-52.73) | -5.72 (-6.05 to -5.38)^*^ |
| Andean Latin America | 23646 (17128-32154) | 447.71 (324.30-608.80) | 5723 (4047-7521) | 92.98 (65.74-122.18) | -5.25 (-6.25 to -4.23)^*^ |
| Central Latin America | 171620 (104447-241239) | 745.59 (453.77-1048.05) | 30312 (17793-42774) | 150.88 (88.56-212.91) | -4.99 (-5.72 to -4.25)^*^ |
| Tropical Latin America | 147922 (93379-204350) | 866.09 (546.74-1196.49) | 43655 (27199-61472) | 253.70 (158.06-357.24) | -4.48 (-7.22 to -1.66)^*^ |
| Southern Latin America | 10199 (7143-13526) | 198.16 (138.78-262.80) | 1923 (1287-2738) | 44.94 (30.09-63.99) | -4.51 (-5.96 to -3.03)^*^ |
| Caribbean | 16999 (10939-23872) | 411.46 (264.78-577.81) | 6089 (3900-8660) | 157.41 (100.83-223.87) | -2.93 (-3.23 to -2.63)^*^ |
| Eastern Sub-Saharan Africa | 828577 (543522-1111297) | 2296.09 (1506.17-3079.54) | 449529 (304088-605398) | 704.63 (476.65-948.95) | -3.77 (-4.03 to -3.51)^*^ |
| Southern Sub-Saharan Africa | 144431 (98116-191935) | 1932.73 (1312.96-2568.42) | 32197 (21412-42874) | 401.01 (266.68-533.98) | -4.99 (-5.52 to -4.45)^*^ |
| Western Sub-Saharan Africa | 1251210 (843990-1660185) | 3500.44 (2361.18-4644.61) | 1006761 (690930-1339806) | 1259.11 (864.12-1675.64) | -3.22 (-3.63 to -2.81)^*^ |
| North Africa and Middle East | 567547 (380575-782777) | 1107.85 (742.88-1527.98) | 116522 (82157-153613) | 190.59 (134.38-251.26) | -5.61 (-6.01 to -5.21)^*^ |
| Central Sub-Saharan Africa | 418211 (271570-563805) | 4027.30 (2615.18-5429.35) | 347916 (241351-480407) | 1651.51 (1145.66-2280.43) | -2.86 (-3.00 to -2.73)^*^ |

**Supplementary Table 3.** Age-standardized prevalence of HIV, syphilis and HBV infection in WCBA by age group in 1990 and 2021, and average annual percentage changes from 2019 to 2021

|  | prevalence |  |
| --- | --- | --- |
|  | ASR (per 100000 population, 2021) | AAPC, 2019-2021 |
| HIV |  |  |
| 15-19 years | 222.09 (202.92-250.83) | 0.76 (0.44 to 1.09)^*^ |
| 20-24 years | 480.65 (442.26-529.32) | 1.34 (1.10 to 1.57)^*^ |
| 25-29 years | 832.43 (780.55-900.84) | 2.36 (2.14 to 2.58)^*^ |
| 30-34 years | 1057.43 (1000.85-1124.42) | 3.89 (3.64 to 4.13)^*^ |
| 35-39 years | 1210.80 (1159.60-1267.01) | 5.50 (5.24 to 5.77)^*^ |
| 40-44 years | 1215.72 (1155.01-1275.90) | 6.19 (6.00 to 6.37)^*^ |
| 45-49 years | 1039.13 (984.34-1086.70) | 6.18 (6.04 to 6.33)^*^ |
| syphilis |  |  |
| 15-19 years | 789.02 (561.51-1107.47) | 0.37 (0.25 to 0.50)^*^ |
| 20-24 years | 1365.58 (766.48-2122.24) | 0.19 (0.11 to 0.27)^*^ |
| 25-29 years | 1610.59 (957.05-2532.29) | 0.07 (-0.14 to 0.28) |
| 30-34 years | 1267.87 (775.26-1957.05) | -0.17 (-0.39 to 0.05) |
| 35-39 years | 928.24 (569.86-1428.60) | -0.00 (-0.17 to 0.17) |
| 40-44 years | 694.04 (430.51-1084.70) | -0.03 (-0.11 to 0.04) |
| 45-49 years | 547.78 (338.66-864.53) | -0.28 (-0.45 to -0.11)^*^ |
| HBV infection |  |  |
| 15-19 years | 2396.19 (2123.70-2719.36) | -2.78 (-2.92 to -2.64)^*^ |
| 20-24 years | 3261.28 (2901.80-3679.85) | -1.94 (-2.05 to -1.83)^*^ |
| 25-29 years | 4045.54 (3604.22-4542.56) | -1.10 (-1.18 to -1.02)^*^ |
| 30-34 years | 4362.31 (3894.28-4871.67) | -0.70 (-0.74 to -0.65)^*^ |
| 35-39 years | 4225.12 (3785.29-4691.53) | -0.84 (-0.89 to -0.79)^*^ |
| 40-44 years | 4124.71 (3705.91-4560.52) | -0.68 (-0.73 to -0.62)^*^ |
| 45-49 years | 4119.78 (3728.81-4537.05) | -0.63 (-0.71 to -0.55)^*^ |


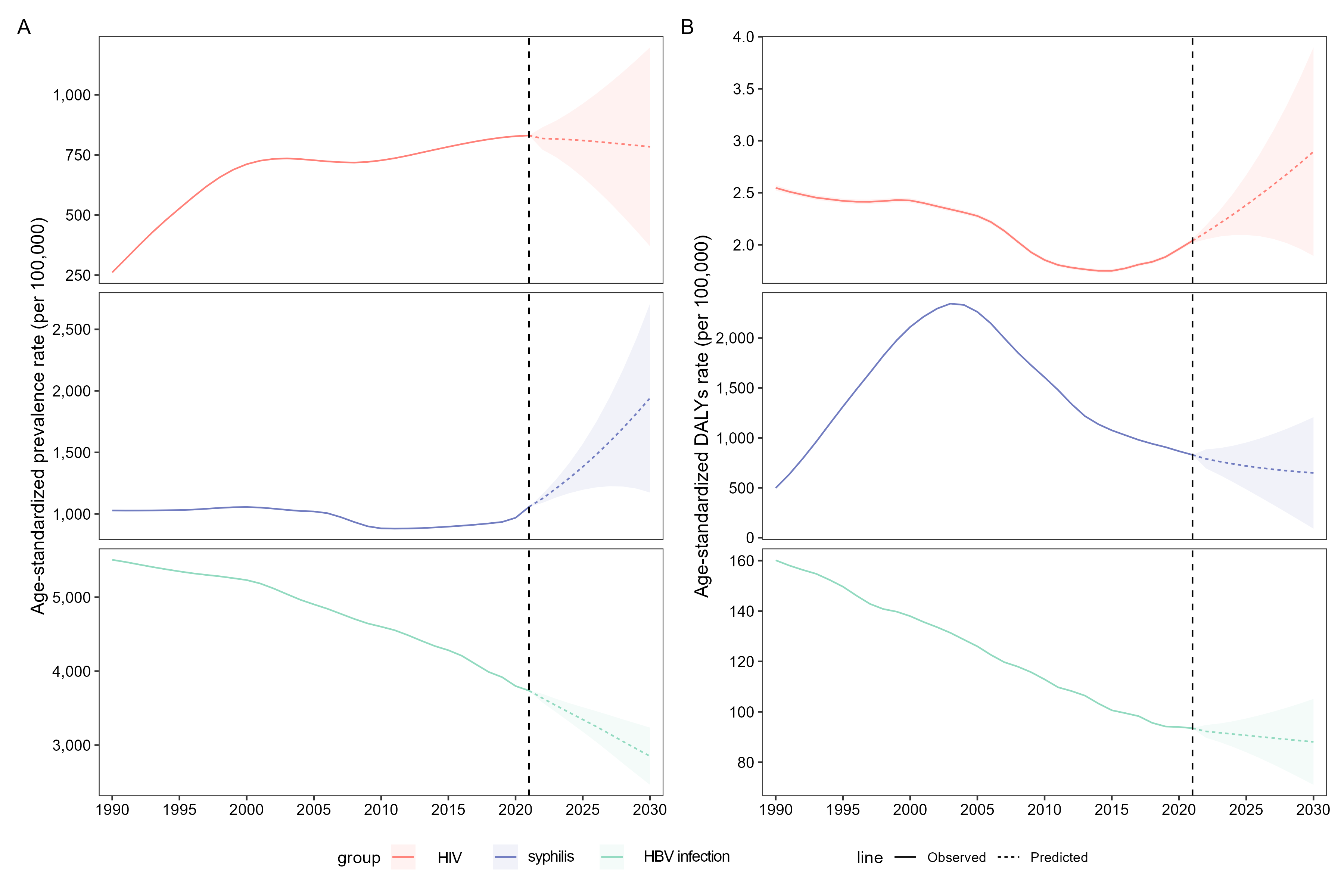


**Supplementary Figure 4.** Statitics and prediction of the ASPR and DALYs rates in all age groups by BAPC model.
